# Biomechanical properties of the bone during implant placement

**DOI:** 10.1186/s12903-021-01442-1

**Published:** 2021-02-25

**Authors:** Ádám László Nagy, Zsolt Tóth, Tamás Tarjányi, Nándor Tamás Práger, Zoltán Lajos Baráth

**Affiliations:** 1 Department of Prosthodontics, Faculty of Dentistry, University of Szeged, Tisza Lajos Krt. 64-64, 6720 Szeged, Hungary; 2 Department of Experimental Dentistry and Oral Biology, Faculty of Dentistry, University of Szeged, Szeged, Hungary

**Keywords:** Biomechanics, Dental implant(s), Fixed and removable prosthodontics, Implant dentistry/implantology, Jaw biomechanics, Oral and maxillofacial surgery

## Abstract

**Background:**

In this research the biomechanical properties of a bone model was examined. Porcine ribs are used as experimental model. The objective of this research was to investigate and compare the biomechanical properties of the bone model before and after implant placement.

**Methods:**

The bone samples were divided in three groups, Group 1 where ALL-ON-FOUR protocol was used during pre-drilling and placing the implants, Group 2 where ALL-ON-FOUR protocol was used during pre-drilling, and implants were not placed, and Group 3 consisting of intact bones served as a control group. Static and dynamic loading was applied for examining the model samples. Kruskal–Wallis statistical test and as a post-hoc test Mann–Whitney U test was performed to analyze experimental results.

**Results:**

According to the results of the static loading, there was no significant difference between the implanted and original ribs, however, the toughness values of the bones decreased largely on account of predrilling the bones. The analysis of dynamic fatigue measurements by Kruskal–Wallis test showed significant differences between the intact and predrilled bones.

**Conclusion:**

The pre-drilled bone was much weaker in both static and dynamic tests than the natural or implanted specimens. According to the results of the dynamic tests and after a certain loading cycle the implanted samples behaved the same way as the control samples, which suggests that implantation have stabilized the skeletal bone structure.

## Background

With the development of dentistry, the aesthetic and functional expectations of patients are also increasing. They anticipate fixed dentures even in total edentulous state. These expectations are challenging for the dentist, especially in cases with severe atrophy of the alveolar ridge, which is particularly complicated, when the teeth have been extracted long time ago. The possible treatment options which allow us to deliver fixed implant-supported dental prosthesis and to achieve a high degree of patient satisfaction, requires to utilize the remaining bone in the most efficient way possible in view of the severity of the involution. The implant placement is usually impossible without guided regeneration surgery [1] in case of elderly people, who typically have D1 quality bone with *high degree of* cortical bone volume [2]. The guided bone regeneration procedure [3] carries high risk of patient morbidity and complications. To avoid the extensive bone augmentation procedure [4, 5] due to the advanced involution, the ALL-ON-FOUR protocol was introduced by Maló [5, 6]. According to this concept, the fixed and immediately loaded prosthesis is supported by four implants in the anterior part of the complete edentulous jaw. The two posterior implants are placed in the interforaminal region, angled, to minimize the cantilever length; the two anterior placed axially, parallel to each other [7]. Both finite analysis and retrospective studies [5] suggest that implants placed this way could be a good alternative, which can safely support the fixed dentures. No clinically significant differences in success rates were found between these methods [8].

An idea presents itself that the mechanical properties of the mandible could be affected by the procedure of pre-drilling and then, substituting the space with a different characteristic material. In this study the possibility that drilling and implant placement could weaken the jawbone against masticatory forces was examined. If this process affects the biomechanical properties, the possibility of three-dimensional torsion deformation of the mandible has to be considered [9, 10]. It was also investigated whether it represents a risk of pathological fractures for the patient, considering the fact that the implants placed with ALL-ON-FOUR protocol are being immediately loaded with the provisional or definitive full-arch prosthesis in 48 h after surgery [5, 11]. The possibility of these deformations and micromovements can be recognized as a deleterious phenomenon during osseointegration [12], however, according to the experimental models of several authors, these micromovements were not proven to be harmful [13].

The basic hypothesis is that the implant placement weakens the biomechanical properties of the bone structure. Our objective is to investigate and compare the mechanical properties of the ribs, before and after implant placement.

## Methods

Fresh, non-frozen, young domestic porcine ribs with soft parts (periosteum, attached muscles, fascia, fat) were obtained from an abattoir. The excess soft parts were removed with a sharp scalpel, however care was taken to ensure that the periosteum was left intact. The main reason for the selection of porcine ribs was the excellent homogeneity and thickness of cortical bone [14] which is similar to a human mandible [15, 16]. The animals were not sacrificed for the purpose of the experiment. The dimensions of the ribs were measured with an analog dial caliper (0.01 mm, Hoffmann Gruppe AK600203). The average length, width and height of the bones were 117.1 mm, 13.4 mm and 9.8 mm, respectively. The average value and standard error of the cortical bone thickness was 2.13 mm ± 0.08 mm. The porcine ribs were randomly divided into three groups. In the first group (Group 1, n = 17) the implants were placed according to the ALL-ON-FOUR protocol: two implants were placed parallel medially (ICX TEMPLANT 4.1 mm × 10 mm, WS-75L surgical contra-angle handpiece, Implantmed Classic SI-923 physiodispenser, W&H, Bürmoos, Austria), and two tilted implants were inserted laterally (ICX TEMPLANT 4.1 mm × 15 mm). In the second group (Group 2, n = 16) the nests of the implants were pre-drilled (WS-75L surgical contra-angle handpiece, Implantmed Classic SI-923 physiodispenser, W&H, Bürmoos, Austria) for the same type and size of implant, but left empty without implant placement. During pre-drilling and placing the implants, the manufacturer’s recommendations and the rules of the profession were kept in mind. No intervention was taken on the ribs in the control group (Group 3 n = 18).

For the mechanical testing, each group was randomly divided into two parts. Half of the samples were tested with a static tensile and compression materials testing machine /Tinnius Olsen H5KT Atec, USA/, while the other half were placed under fatigue test by an All-*Electric* Dynamic Test Instrument (Instron ElectroPuls™ E3000, USA) [17].

**Fig. 1:**
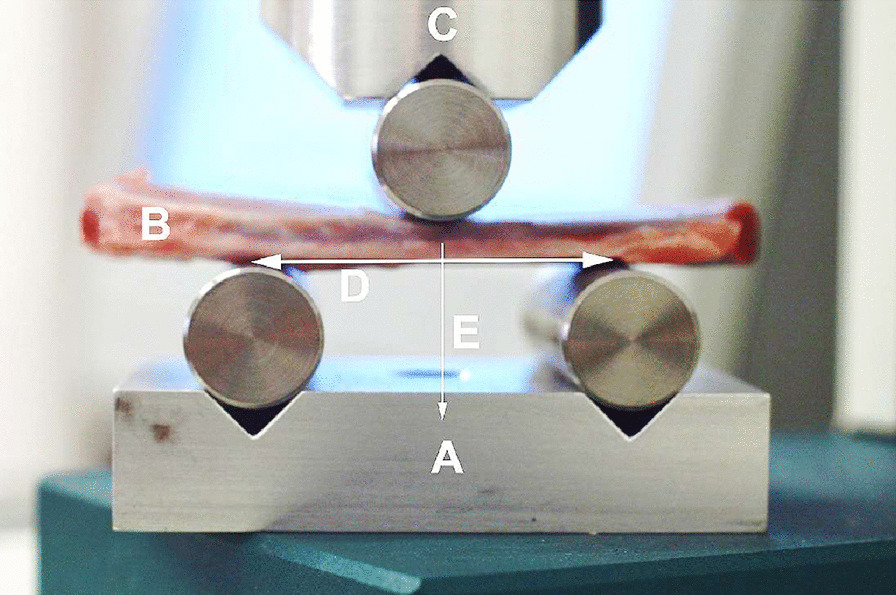
Experimental layout. **a** Supporting platform with point support rollers; **b** pork rib segment; **c** pressure head of the mechanical tear / break device with the roller used for point loading. **d** distance between support points (standard 40 mm). **e** vector of the force acting on the bone segment

For the mechanical testing 3-point bending tests were performed, which are most widely accepted for fracture testing [18–21]. Mechanical components were manufactured individually that could be applied for both the static and dynamic equipment. The devices thus became suitable for performing 3-point bending tests (Fig. 1).

During the static load measurements, the bending deformation was increasing steadily on the bones. The according force was measured, digitized. The equipment recorded the position of the crosshead and the measured force. The maximum deformation was 10 mm, which was reached in 5 s. During the measurement an automatic halt was actuated, when the device observed a sudden decrease in the force.

The other part of the samples was examined by a dynamic fatigue test. The dynamic test followed the arrangement of three point bending fatigue measurements. [22, 23]. Prior to the dynamic tests, the stiffness of each rib was determined by measuring the force–deflection curve between 0.2 and 0.8 mm deflection. After this process the fatigue test was performed on the samples, where the initial deflection was set to 2 mm, which was reached in 5 s. The fatigue test was performed in deflection control mode. The fatigue signal was a sinus function with 20 Hz frequency at 0.5 mm deflection amplitude over 10.000 cycle. At the end of the fatigue process the load was decreased to 0 N in 5 s.

Shapiro–Wilk test was performed to validate the normality of distribution of the measured data. Kruskal–Wallis non-parametric test was used to compare the different groups’ measured force values and as post-hoc tests the Mann–Whitney U non-parametric statistical tests were used. The significance level in these tests were set to 5% (p < 0.05). SPSS statistical software (version 25; IBM Co., Armonk, NY, USA) was used for statistical analysis.

## Results

### Results of the static load test

**Fig. 2:**
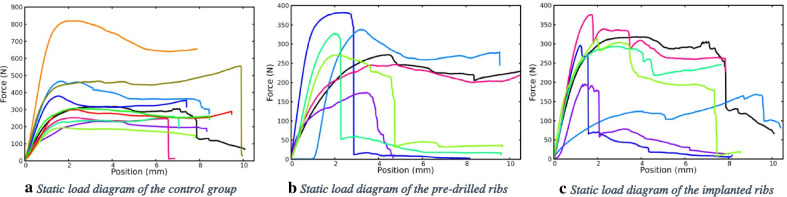
Measurement results of the static load tests. **a** Static load diagram of the control group. **b** Static load diagram of the pre-drilled ribs. **c** Static load diagram of the implanted ribs

**Fig. 3:**
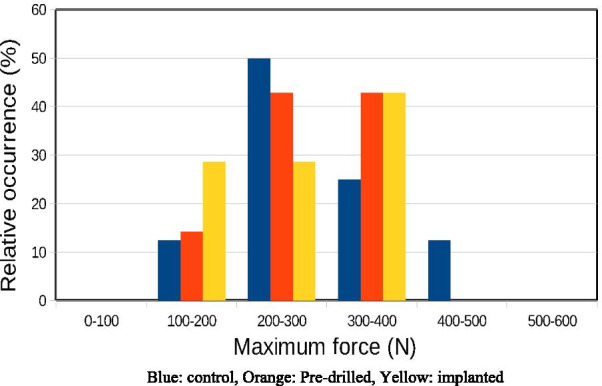
The occurrence of the maximum static load force values: Blue: control, Orange: Pre-drilled, Yellow: implanted

The graph in Fig. 2 shows the measurement results of the static load tests: the first stage of the load–deflection curve can be described as an almost straight increasing line, which represents the flexible range of the rib. After the maximum force exerted, even a smaller force was sufficient for further deflection. Figure 3 shows the occurrence of the measured maximum force ranges: during the load the measured average forces values were higher on the control samples than on the drilled bones The mean of the maximum force (and standard error) for the control samples was 298.9 ± 30.95 N, for the pre-drilled was 287.1 ± 25.93 N and 280.29 ± 27.51 N for the implanted group. We found no statistically significant difference between the groups (p = 0.979).

The area under the curves on the diagrams of Fig. 2 (S) describes a quantity, which correlates with the toughness of the ribs, and can be calculated with the following formula:

$$S = \int\limits_{0}^{x1} {F(x)dx}$$

**Fig. 4:**
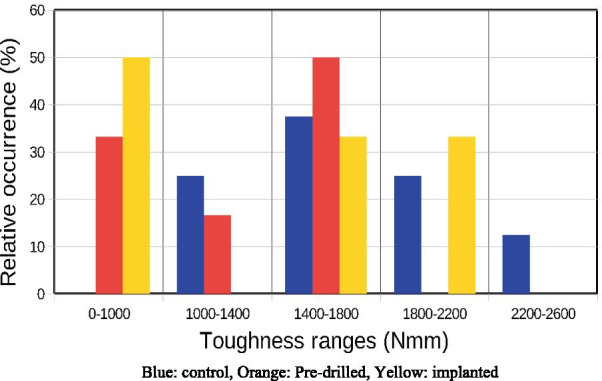
Occurrence of various toughness ranges in the study groups. Blue: control, Orange: Pre-drilled, Yellow: implanted

Figure 4 shows the S values in Nmm registered during the test.

Mean S value was 1701.37 ± 166.335 Nmm in the control, 1175.77 ± 128.832 Nmm in the pre-drilled and 1235.56 ± 248.392 Nmm in the implanted group. There are no significant differences between the groups in the calculated S toughness related values (p = 0.16, Kruskal–Wallis test).

### Results of the dynamic fatigue test

**Fig. 5:**
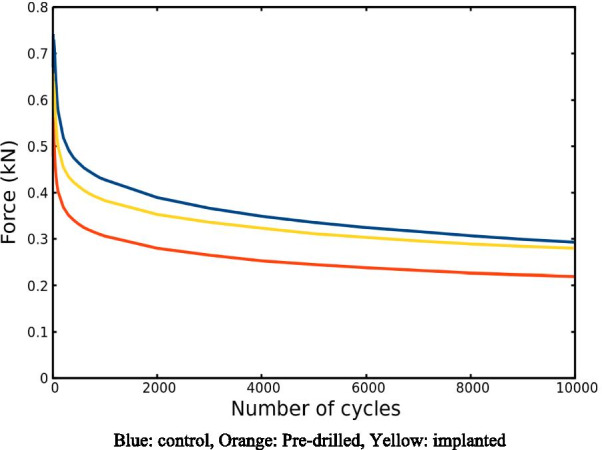
The force values measured for maximum deformation (2,5 mm) depending on the number of cycles Blue: control, Orange: Pre-drilled, Yellow: implanted

To analyze the results of the dynamic fatigue tests the Kruskal–Wallis statistic test (control vs. 1, 2) was performed on the measured force values measured for maximum deflection (2.5 mm) at specified times (100th, 2000th, 9000th cycles). The results are shown in Fig. 5.

At the 100th cycle the average measured force values were: 0.5766 ± 0.033 kN in the control, 0.4030 ± 0.081 kN, in the pre-drilled, 0.4991 ± 0.073 kN in the implanted group. The statistical test showed significant differences for the measured values between the groups (p = 0.014, Kruskal–Wallis test).

At the 2000th cycle the average measured force values were: 0.3896 ± 0.027 kN in the control, 0.2800 ± 0.056 kN, in the pre-drilled, 0.3530 ± 0.049 kN in the implanted group. The statistical test showed a significant difference for the measured values between the groups (p = 0.015, Kruskal–Wallis test).

At the 9000th cycle the average values were: 0.2999 ± 0.015 kN in the control, 0.2227 ± 0.042 kN, in the pre-drilled, 0.2840 ± 0.042 kN in the implanted group. The statistical test showed a significant difference for the measured values between the groups (p = 0.026, Kruskal–Wallis test).

The difference between the groups was tested with Mann–Whitney test. This showed a significant difference in the measured force values between the control and drilled ribs 100th cycle (p = 0.001, Mann–Whitney U test), which difference remains consistent at the 2000th cycle (p = 0.002, Mann–Whitney U test) and 9000th cycle (p = 0.005, Mann–Whitney U test).

The measured force values in any of the cycles examined showed no statistical significant difference between the control and the implanted group (100th cycle p = 0.243, 2000th cycle p = 0.447, 9000th cycle p = 0.72, Mann–Whitney U test), furthermore, the summary graph (Fig. 5) shows that they exhibit very similar force values from cycle 9000.

No significant difference was found between the drilled and implanted ribs in the post-hoc test at 100th cycle (p = 0.33, Mann–Whitney U test) 2000th cycles (p = 0.136, Mann–Whitney U test), at 9000th cycles (p = 0.094, Mann–Whitney U test).

## Discussion

The purpose of this study was to examine and discuss the deterioration of bone mechanical properties as a function of bending forces before and after implant placement in order to seek an answer to the question, whether implant placement can weaken the bone structure.

The three-point bending tests, reported in the literature, were performed only with intact bones [21, 28, 29] and not pre-drilled and implanted ones, as in this work.

Static load tests showed significant differences between the groups tested. In the case of intact bone samples, the load curves shown in Fig. 2 are continuous, and the sudden reduction in force associated with fractures is observed only over a large deformation of ~ 6.6 mm. The maximum force observed for the intact bones is in the range of 200–800 N with an average maximum force of 299 ± 31 N. Typically, the maximum force values were achieved with 1.5 to 3 mm deflection.

For the drilled samples, the resistance force maximum (170–390 N) decreased relative to the control samples, which is well observed in Fig. 2. Most measurements show single or gradual fractures in the 2.4–5 mm deflection range, well below the damage limit of the intact bones. The maximum force observed was 287 ± 26 N. The reduction of the damage limit clearly indicates the weakening of the bone’s resistance to force, which is partly due to the decrease in the effective bone thickness in the drilled region.

According to our static load tests, filling the pre-drilled nest with implants did not improve the mechanical resistance of the bones. For the implanted samples the maximum force measured was in the range 175–380 N, the mean maximum force decreased to 280 ± 28 N. The deflection values corresponding to the first partial fracture are in the range of 1.6–4.5 mm, which is smaller compared to the intact and drilled bone values. Partial cracks were observed between the two middle implants during the load. The appearance of a crack was often accompanied by a sound effect. The earlier cracks appear to be due to the fact that the holes are filled with harder material than the spongy bone, consequently local stresses at the implant-bone interface are exerted during loading.

If the local stress value is greater than the strength of the cortical bone, a crack appears [24], but the macroscopic fracture of the bone does not occur [25]. As the deflection increases, the force–deflection curve shows small breaks, indicating the appearance of new cracks. The local fractures provide stress relaxation, resulting in a higher deflection values for appearance of macroscopic fracture at 7.3–9.5 mm compared to the drilled bone. Due to this phenomenon, the toughness of the implanted specimens will be higher than that of the drilled specimens.

For fatigue tests, the same temporal function of deflection was applied throughout the experiments. To achieve the same deflection at a higher cycle number, a lower force was required for each sample, as shown in Fig. 5. Initially, the decrease in the force values is greater, and with higher cycle numbers, the reduction of the force slows down. This phenomenon shows the weakening of the mechanical structure due to bending cycles. Each cycle causes reduction of bone stiffness [26]. However, macroscopic fractures did not occur at the set deflection values and cycle numbers.

For all fatigue tests, the force required for a pre-set deflection was the highest for intact bone and the lowest for drilled bone. This significant weakening is due to the reduction of local bone volume.

In the case of implanted bones, the maximum force values for a given deflection are between the values of the intact and the drilled bone. Initially, the difference compared to intact bone is greater, but with a higher number of cycles this difference disappears.

Overall, the results of our mechanical examinations showed that the placement of the holes in the bone significantly reduces the stiffness and mechanical strength of the bone, which leads to the appearance of macroscopic fractures even at smaller deformations. The implants partially restore the integrity of the bone and increase the load-bearing capacity against the macroscopic fracture compared to the drilled samples. However, the implanted bone does not reach the mechanical strength of intact bone.

This topic was explored by finite element analysis, and many studies have been conducted on the relationship between the bone and implants under the All-on-four protocol. According to Sannino, distal implants placed at 15, 30, and 45 degrees, with a greater angle at the implant-bone interface, exert the greatest stress, but this mechanical stress value is still lower than what the implant and bone can withstand [27].

Our static load result shows that the toughness is less in the case of drilled bones but not statistically significant. The measured maximum force values also showed no statistically significant difference during the static load. However, during the fatigue load the drilled bones showed significant difference compared to the control samples. The control and the ALL-ON-FOUR implanted samples showed very similar measured force values after the 9000th cycle.

It is important to note that these measurements were performed on non-osseointegrated samples. In the event when osseointegration occurs, mechanical properties are expected to improve further. However, our experiment shows that local mechanical stresses appear at the bone-implant interface, which reduces the force required to cause fractures. A limitation of our study is that the bending forces applied in the tests occur only in extreme cases in clinical circumstances. However, the cyclicity and the magnitudes were in accordance with physiologically observable chewing movements. A further limitation of our research is that the applied protocol does not allow the implant-bone interface to be investigated in a direct way, unlike with the finite element analysis tests.

## Conclusion

With the limitations of this in vitro ALL-ON-FOUR study, the pre-drilled bone was much weaker in both static and dynamic tests than the natural or implanted specimens. According to the results of the dynamic tests and after a certain loading cycle the implanted samples behaved the same way as the control samples, which suggests that implantation have stabilized the skeletal bone structure.

## Data Availability

The datasets used and/or analysed during the current study available from the corresponding author on reasonable request.

## References

[1] Clavero J, Lundgren S (2003). Ramus or chin grafts for maxillary sinus inlay and local onlay augmentation: comparison of donor site morbidity and complications. Clin Implant Dent Relat Res.

[2] Trisi P, Rao W (1999). Bone classification: clinical-histomorphometric comparison. Clin Oral Implants Res.

[3] Kuchler U, von Arx T (2014). horizontal ridge augmentation in conjunction with or prior to implant placement in the anterior maxilla: a systematic review. Int J Oral Maxillofac Implants.

[4] Urban IA, Monje A (2019). Guided bone regeneration in alveolar bone reconstruction. Oral Maxillofac Surg Clin North Am.

[5] Malo P, Rangert B, Nobre M (2003). “All-on-Four” immediate-function concept with Branemark System implants for completely edentulous mandibles: a retrospective clinical study. Clin Implant Dent Relat Res.

[6] Rasouli R, Barhoum A, Uludag H (2018). A review of nanostructured surfaces and materials for dental implants: surface coating, patterning and functionalization for improved performance. Biomater Sci.

[7] Bevilacqua M, Tealdo T, Pera F, Menini M, Mossolov A, Drago C (2008). Three-dimensional finite element analysis of load transmission using different implant inclinations and cantilever lengths. Int J Prosthodont.

[8] Malo P, de Araujo NM, Rangert B (2007). Short implants placed one-stage in maxillae and mandibles: a retrospective clinical study with 1 to 9 years of follow-up. Clin Implant Dent Relat Res.

[9] Daegling DJ, Hylander WL (1998). Biomechanics of torsion in the human mandible. Am J Phys Anthropol.

[10] Seong W-J, Kim U-K, Swift JQ, Heo Y-C, Hodges JS, Ko C-C (2009). Elastic properties and apparent density of human edentulous maxilla and mandible. Int J Oral Maxillofac Surg.

[11] Maló P, de Araujo NM, Lopes I (2008). A new approach to rehabilitate the severely atrophic maxilla using extramaxillary anchored implants in immediate function: a pilot study. J Prosthet Dent.

[12] Sugiura T, Yamamoto K, Horita S, Murakami K, Tsutsumi S, Kirita T (2017). Effects of implant tilting and the loading direction on the displacement and micromotion of immediately loaded implants: an in vitro experiment and finite element analysis. J Periodontal Implant Sci.

[13] Kourtis LC, Carter DR, Beaupre GS (2014). Improving the estimate of the effective elastic modulus derived from three-point bending tests of long bones. Ann Biomed Eng.

[14] Friberg B, Sennerby L, Roos J, Johansson P, Strid CG, Lekholm U (1995). Evaluation of bone density using cutting resistance measurements and microradiography: an in vitro study in pig ribs. Clin Oral Implants Res.

[15] Kim S-J, Yoo J, Kim Y-S, Shin S-W (2010). Temperature change in pig rib bone during implant site preparation by low-speed drilling. J Appl Oral Sci.

[16] Szalma J, Lovász BV, Vajta L, Soós B, Lempel E, Möhlhenrich SC (2019). The influence of the chosen in vitro bone simulation model on intraosseous temperatures and drilling times. Sci Rep.

[17] Lee S-W, Kim S-G (2014). Membranes for the Guided Bone Regeneration. Maxillofac Plast Reconstr Surg.

[18] Horita S, Sugiura T, Yamamoto K, Murakami K, Imai Y, Kirita T (2017). Biomechanical analysis of immediately loaded implants according to the “All-on-Four” concept. J Prosthodont Res.

[19] Jiang F, Rohatgi A, Vecchio KS, Cheney JL (2004). Analysis of the dynamic responses for a pre-cracked three-point bend specimen. Int J Fract.

[20] Leppanen O, Sievanen H, Jokihaara J, Pajamaki I, Jarvinen TLN (2006). Three-point bending of rat femur in the mediolateral direction: introduction and validation of a novel biomechanical testing protocol. J Bone Miner Res.

[21] Ayagara AR, Langlet A, Hambli R (2019). On dynamic behavior of bone: experimental and numerical study of porcine ribs subjected to impact loads in dynamic three-point bending tests. J Mech Behav Biomed Mater.

[22] Sadeghi H, Espino DM, Shepherd DET (2017). Fatigue strength of bovine articular cartilage-on-bone under three-point bending: the effect of loading frequency. BMC Musculoskelet Disord.

[23] Mori S, Burr DB (1993). Increased intracortical remodeling following fatigue damage. Bone.

[24] Zioupos P, Hansen U, Currey JD (2008). Microcracking damage and the fracture process in relation to strain rate in human cortical bone tensile failure. J Biomech.

[25] Vashishth D, Tanner KE, Bonfield W (2003). Experimental validation of a microcracking-based toughening mechanism for cortical bone. J Biomech.

[26] Keaveny TM, Wachtel EF, Kopperdahl DL (1999). Mechanical behavior of human trabecular bone after overloading. J Orthop Res.

[27] Sannino G (2015). All-on-4 concept: a 3-dimensional finite element analysis. J Oral Implantol.

[28] Jamsa T, Jalovaara P, Peng Z, Vaananen HK, Tuukkanen J (1998). Comparison of three-point bending test and peripheral quantitative computed tomography analysis in the evaluation of the strength of mouse femur and tibia. Bone.

[29] Turkozan NY, Mammadov C (2011). Biomechanical properties of the body and angle of the sheep mandible under bending loads. Dent Traumatol.

